# Artificially Intelligent Nanoarray Detects Various Cancers by Liquid Biopsy of Volatile Markers

**DOI:** 10.1002/adhm.202200356

**Published:** 2022-07-14

**Authors:** Reef Einoch Amor, Assaf Zinger, Yoav Y. Broza, Avi Schroeder, Hossam Haick

**Affiliations:** ^1^ Department of Chemical Engineering and Russell Berrie Nanotechnology Institute Technion – Israel Institute of Technology Haifa 3200003 Israel; ^2^ Laboratory for Targeted Drug Delivery and Personalized Medicine Technologies Department of Chemical Engineering Technion – Israel Institute of Technology Haifa 3200003 Israel

**Keywords:** breast cancer, liquid biopsies, machine‐ learning, nanotechnology, ovarian cancer, pancreatic cancer, sensors

## Abstract

Cancer is usually not symptomatic in its early stages. However, early detection can vastly improve prognosis. Liquid biopsy holds great promise for early detection, although it still suffers from many disadvantages, mainly searching for specific cancer biomarkers. Here, a new approach for liquid biopsies is proposed, based on volatile organic compound (VOC) patterns in the blood headspace. An artificial intelligence nanoarray based on a varied set of chemi‐sensitive nano‐based structured films is developed and used to detect and stage cancer. As a proof‐of‐concept, three cancer models are tested showing high incidence and mortality rates in the population: breast cancer, ovarian cancer, and pancreatic cancer. The nanoarray has >84% accuracy, >81% sensitivity, and >80% specificity for early detection and >97% accuracy, 100% sensitivity, and >88% specificity for metastasis detection. Complementary mass spectrometry analysis validates these results. The ability to analyze such a complex biological fluid as blood, while considering data of many VOCs at a time using the artificially intelligent nanoarray, increases the sensitivity of predictive models and leads to a potential efficient early diagnosis and disease‐monitoring tool for cancer.

## Graphical abstract

Early cancer detection can immensely improve prognosis. Here, a potential early diagnosis and disease‐monitoring tool is proposed based on volatile organic compound (VOC) patterns in the blood headspace. An artificial intelligence nanoarray in combination with machine learning models gives high performances for early cancer (>84% accuracy) and metastasis (>97% accuracy) detection.

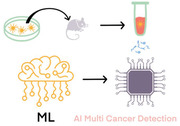

## 1 Introduction

Cancer is quickly becoming the greatest health hazard of our days. Over 19.3 million new cancer cases were diagnosed in 2020 worldwide. Cancer is usually not symptomatic in its early stages,^[^ ^1^, ^2^ ^]^ so localized tumors can only be found through widespread screening of the population.^[^ ^3^, ^4^ ^]^ The effectiveness of cancer screening varies with the cancer site and available techniques. For example, the prognosis of breast cancer (BC) patients is relatively reasonable and improves if the disease is discovered early.^[^ ^5^ ^]^ Mammography finds tumors at an early stage (even in situ carcinoma) when the disease responds well to available therapies, and the costs (≈$100 per test) are moderate. However, mammography uses X‐rays, which can cause radiation‐induced mutations, and the image quality depends on the breast's structure.^[^ ^6^, ^7^ ^]^ Not like BC, pancreatic cancer (PC) is hard to diagnose at its early stage, where most cases are found to be already metastatic at initial diagnosis. Therefore, it is considered one of the deadliest cancers with poor outcomes.^[^ ^8^ ^]^ These poor survival rates have not changed significantly in nearly 40 years.^[^ ^9^, ^10^ ^]^ Similarly, ovarian cancer (OVC) is the second most common and the most lethal gynecologic malignancy in the western world. There are no screening tests forOVC and with diagnosis often in the late stages; recurrence is high and, consequently, has a terrible prognosis.^[^ ^11^, ^12^, ^13^ ^]^

During tumor formation and progression, treatment decisions are commonly made based on diagnostic routine testing of primary tumor samples. Even though biopsy is still the gold standard diagnostic tool, it suffers from many disadvantages. It is invasive, expensive, and requires highly trained personnel.^[^ ^14^, ^15^ ^]^ Moreover, it is not accurate enough for personalized treatment and treatment monitoring since tumor biopsies eventually result in only a fragment of the tumor and therefore provides just limited information on its heterogeneity, and the genetic and epigenetic variations of a patient's cancer. This is in addition to genetic and epigenetic changes formed in the tumor during its manifestation, adding to the complexity of decision‐making.^[^ ^16^, ^17^, ^18^ ^]^ Since it is improbable that a patient will undergo consecutive biopsies of both primary and metastatic lesions along with disease progression, other methods have been developed.

An emerging approach for early detection is based on gene and/or protein analysis. By determining cancer risk, prognosis, and targeted (or personalized) therapy, this approach may improve clinical outcomes for cancer patients.^[^ ^19^, ^20^, ^21^, ^22^ ^]^ Advancement in identifying biomarkers, mutations, and genomic signatures far outstrip the slow development in treatments based on these molecular advances.^[^ ^23^, ^24^, ^25^ ^]^ The main difficulties in developing novel and efficient biomarkers comprise: tumor heterogeneity, the highly complex interplay between the environment and host, and the complexity, diversity, and redundancy of tumor‐cell signaling networks involving genetic, epigenetic, and microenvironmental effects.^[^ ^26^, ^27^, ^28^, ^29^, ^30^, ^31^ ^]^ Blood tests have been proposed but show moderate accuracy in many diseases (e.g., lung cancer (LC)) and have not been validated for clinical use, to date. Biomarker analysis for medical applications shows great promise, but the combination of simultaneous biomarker analysis from different sample matrices has not yet been utilized.^[^ ^32^, ^33^, ^34^ ^]^ Indeed, blood tests have failed to show any cost‐effectiveness in screening for LC. However, a few indirect blood markers may suggest possible LC, among them Parathyroid Hormone (PTH), Carcinogenic Antigen (CEA), and Cytokeratin Fragment 19(CYFRA21‐1).^[^ ^35^, ^36^ ^]^

A promising approach that overcomes many of these shortcomings and would have the potential for high accuracy of detection levels is liquid biopsy. Liquid biopsy is a minimally invasive and more sustainable alternative to cross‐examine cancer cells repeatedly and longitudinally. Liquid biopsy refers to the sampling of body fluids, mainly blood and saliva, urine, cerebrospinal fluid, and others. In cancer patients liquid biopsy is mostly used for the isolation of circulating tumor cells (CTCs), circulating tumor DNA (*ct*DNA), as well as additional tumor‐derived bodies (e.g., exosomes), particularly in cases that tumor biopsy is clinically challenging to obtain.^[^ ^14^, ^37^, ^38^, ^39^, ^40^ ^]^ Despite their advantages, those cancer biomarkers are rare, CTCs, for example, have a concentration of 1–10 cells per mL of blood, each of which is surrounded by 6 × 10^6^ leukocytes, 2 × 10^8^ platelets, and 4 × 10^9^ erythrocytes.^[^ ^41^, ^42^ ^]^ This makes their separation, isolation, and real‐time monitoring very challenging, mainly when these processes rely on their physical properties (e.g., size, density, electric charge, staining properties). On the other hand, using antigens for selective detection and classification of those cancer biomarkers remains relatively limited in terms of accuracy.^[^ ^43^, ^44^, ^45^ ^]^

To cope with these challenges, we adopt a different tactic for low‐risk, fast, and minimally invasive diagnostics that signifies a comprehensive approach for identifying and quantifying CTCs and host responses via blood samples. This approach relies on the findings that cancer cells^[^ ^46^, ^47^, ^48^, ^49^ ^]^ and/or their micro‐environment emanate volatile organic compounds (VOCs).^[^ ^50^, ^51^, ^52^, ^53^, ^54^, ^55^, ^56^, ^57^ ^]^ Some of these VOCs could be found in the cancer cell itself, but the others could be found in the blood,^[^ ^58^, ^59^, ^60^ ^]^Therefore, blood chemistry and/or metabolic changes can reflect cancer, rather than tumor imaging or histopathology. For this reason, it is hypothesized that detection of VOCs in blood would increase the probability and accuracy of detection of cancer.^[^ ^61^ ^]^ Relying on this hypothesis, we describe the construction and use of a nanomaterial‐based sensors array coupled with adaptable machine‐learning (ML) for enabling fast, accurate, and easy‐to‐perform liquid biopsy from the blood for both detection and classification of various cancers. The same blood samples were also analyzed with gas chromatography linked with mass spectrometry (GC‐MS) to validate the sensing results, determining the chemical composition and related differences between the examined samples.

## 2 Results

Clinical trials are the preferred choice for confirming the efficiency of the sensors array in cancer diagnosis. Yet, clinical trials are time‐consuming and expensive. Therefore, simpler and more controllable approaches are called for, viz. using animal models that simulate the cancer and healthy states. During the development and/or adaptation of an array of sensors this strategy provides key advantages over the clinical studies, because it can accurately provide the iterative feedback needed during sensor optimization without the interference of confounding parameters, such as, patients’ diet, metabolic state, genetics, etc.

**Figure 1:**
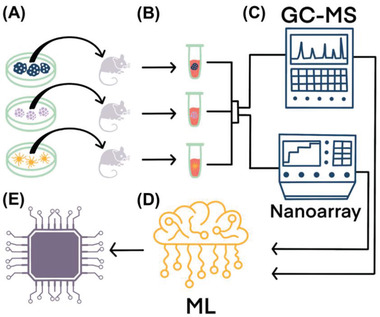
Illustration of the experimental approach. A) PC, BC, and OVC Tumor model formation (see Experimental Section) lead to B) a change in the blood VOCs pattern. C) Exposure of the blood headspace to the sensor‐array with complementary GC‐MS analysis D) combined with ML algorithms can offer high accuracy data on the patient's health. Chemiresistors are based on organically stabilized spherical gold nanoparticles (GNPs) (core diameter 3–4 nm), 2D random networks of single‐walled carbon nanotubes (RN‐SWCNTs) capped with different organic layers, and polymeric composites. E) Potential lab on chip (LoC) for fast, inexpensive, and accurate cancer early detection and monitoring.

**Figure 2:**
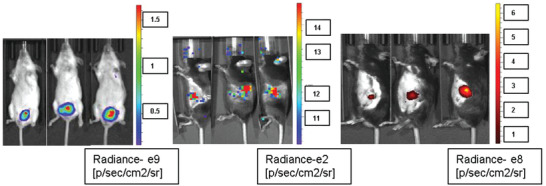
BC, PC, and OVC assessments using IVIS. A) BC tumor model. B) OVC tumor model. C) PC tumor model. All images are acquired by IVIS using the fluorescent or luminescent options.

**Figure** **1** summarizes the design of the study. In the first stage, 4T1 BC, ductal adenocarcinomaPC, and OVC models were established (see method section) and validated using in vivo imaging system (IVIS) spectrum computed tomography (CT) system. In the representative examples (**Figure** **2**), tumor formation for each type of the mentioned cancer can be assessed clearly. We waited 14 days after primary tumor formation for the metastatic BC model and validated the metastases formation by harvesting mice lungs on the day of blood withdrawal. Figure S1, Supporting Information, shows a representative image of lung metastases formed in metastatic BC (M‐BC) model mice.

**Figure 3:**
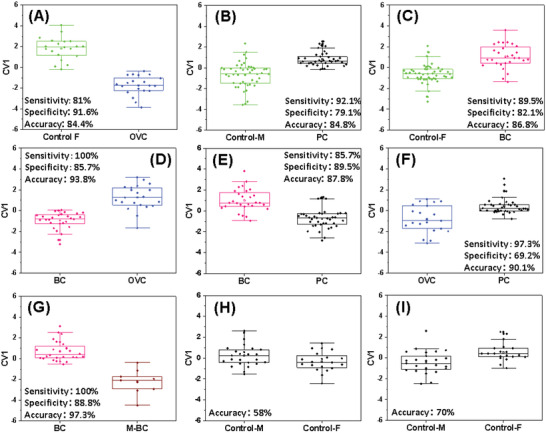
Box plots of the DFA's first canonical variable (CV1) computed from the GNP sensors response to the blood samples headspace originated from mice cancer models. A) Control‐F versus OVC; B) Control‐M versus PC; C) Control‐F versus BC; D) BC versus OVC; E) BC versus PC; F) OVC versus PC; G) BC versus M‐BC. Binary comparison of Male/ Female confounding factor using the combination discriminated between H) BC versus PC and I) OVC versus PC. BC—breast cancer *n* = 36; M‐BC—metastatic breast cancer *n* = 10; OVC—ovarian cancer *n* = 17; PC—pancreatic cancer *n* = 38; F—female *n* = 22; M—male *n* = 34.

In the second stage, the headspace of the blood samples collected from the animal models were analyzed by sensor array composed of chemiresistors based on spherical gold nanoparticles (GNPs) capped with different organic layer, and 2D random networks of single‐walled carbon nanotubes (RN‐SWCNTs) capped with different polymeric composites in conjugation with ML methods (see Experimental Section and Supporting Information for detailed working mechanism).^[^ ^56^, ^57^, ^58^, ^60^, ^62^, ^63^, ^64^, ^65^, ^66^, ^67^, ^68^ ^]^ The rationale behind using two different sensing surfaces is that different transducers (being based on different physical quantities) can provide orthogonal information.^[^ ^58^ ^]^ The GNP sensors are sensitive to molecules adsorbed between adjacent nanoparticles and therefore they excel in the detection of nonpolar VOCs—an important portion of the VOCs clinical samples.^[^ ^48^, ^52^, ^54^, ^55^, ^69^ ^]^ On the other hand, the RN‐ SWCNT sensors are sensitive to polar VOCs, due to carrier donation/withdrawing from/to the RN‐SWCNTs. In a few instances (mainly under high VOC concentrations), these sensors are sensitive to nonpolar VOCs, due to scattering mechanism between adjacent CNTs^[^ ^70^, ^71^, ^72^, ^73^ ^]^ So far, the use of an individual sensor (or feature) of any of these chemistries (see Experimental Section and workflow in Figure S2, Supporting Information) resulted in a medium quality of detection and discrimination. For example, the maximum accuracy for discriminating between cancer and healthy controls was 70% and was similar to those obtained for discriminating between different cancer model samples. In blood samples of primary BC or metastatic BC, the best individual sensor had a maximum accuracy of 75%. To improve classification, signals from multiple sensors, accounting chemistries from the two categories, were combined so that the others provide the data failed to be spotted by a single sensor, and, the imprecision of a single sensor could typically be compensated by similar ones. Indeed, combining data from multiple sensors signals acts as an internal validation test—allowing the system software to reduce temporarily mistaken readings or, preferably, correct them. Therefore, discriminant factor analysis (DFA) from at least three sensors (or features) was used namely the ML process. A complete list of sensors for each model can be found in Table S1, Supporting Information. The discrimination accuracy, as well as the sensitivity and specificity of the classification model, was evaluated based on the output of the samples measured in order to determine the ability of the array to classify correctly the VOC‐samples. Thus, discrimination accuracy between different cancer models and naïve mice varied between 84.4–86.8% (**Figure** **3**A–C). At the same time, the same DFA‐based discrimination ability between different cancer models (BC, OVC, and PC) varied between 87.8–93.8% accuracy based on three features model (Figure 3D–F). A 3‐feature model based on chemiresistive films of GNPs coated with decanethiol and 2‐naphthalenethiol showed 81% sensitivity, 91.6% specificity, and 84.4% accuracy discriminating OVC mice from naïve mice blood samples (Figure 3A). On the other hand, 3‐feature model based on chemiresistive films of RN‐SWCNTs coated with diketopyrrolopyrole‐anthracene (FAF) and GNPs coated with 3‐ethoxythiophenolthiol and 2‐nitro‐4‐(trifluoromethyl)‐benzenethiol showed 92.1% sensitivity, 79.1% specificity, and 84.8% accuracy in discriminating PC mice from naïve mice blood samples (Figure 3B). Our sensor array was also able to discriminate efficiently between different cancer stages, as seen in Figure 3C,G. A Model‐based on chemiresistive films of RN‐SWCNTs coated with diketopyrrolopyrole‐naphthalene (TNT) and GNPs coated with 4‐chlorobenzenemethanethiol and hexanethiol showed 89.5% sensitivity, 82.1% specificity, and 86.8% accuracy in discriminating BC mice from naïve mice blood samples (Figure 3C). A model based on chemiresistive films of RN‐SWCNTs coated with diketopyrrolopyrole‐naphthalene (TNT) and GNPs coated with 2‐ethylhexanethiol octadecanethiol showed 100% sensitivity, 88.8% specificity, and 97.3% accuracy in discriminating BC mice from metastatic BC (M‐BC) mice blood samples (Figure 3G).

**Figure 4:**
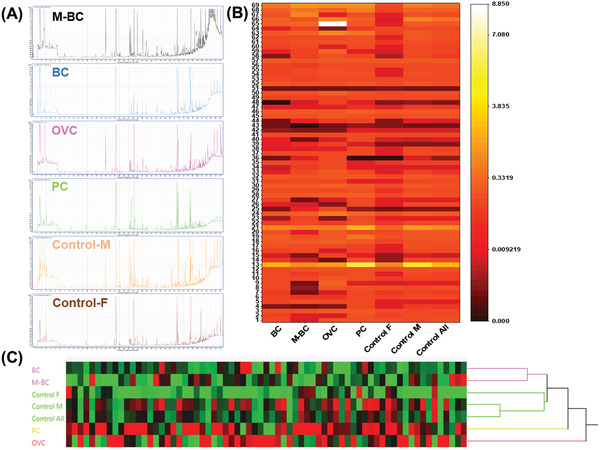
A) Representative chromatograms from (top to bottom) M‐BC, BC, OVC, PC, Control‐M, Control‐F, respectively. B) Mice cancer models VOCs profile colormap. Colormap shows the abundance of VOC profile as found in GC‐MS (red‐low, white‐high) obtained from the headspace of the blood of different mice cancer models. C) hierarchical clustering and colormap of the standardized VOC profiles abundance as found in GC‐MS (red‐high, green‐low). Only VOCs that presented a significant difference among the groups are shown. BC—breast cancer *n* = 36; M‐BC—metastatic breast cancer *n* = 10; OVC—ovarian cancer *n* = 21; PC—pancreatic cancer *n* = 39; F—female *n* = 22; M—male *n* = 36. VOCs tentative identification is given in Table S1, Supporting Information.

To confirm the results of the sensors array, GC‐MS was carried out for the blood samples in the third stage. For comparison, the VOC pattern in naïve mice was examined. The GC‐MS analysis resulted in >200 VOCs in the different samples, yet only 69 VOCs were further selected for examination. These VOCs showed a significant difference between at least one model comparison. Multiple linear regression for each VOC was used to test possible correlation between abundance and the covariates. Representative chromatogram of different groups is presented in **Figure** **4**A showing the different profiles. A significant pattern change is seen in Figure 4B, where each study group shows a different abundance of these 69 VOCs. This pattern may help to discriminate easily between different cancer models. Thus, a hierarchical clustering (using Ward's minimum variance method) was performed on same VOC data and resulted in clear clustering of BC subgroups and control subgroups. At the same time, PC and OVC were separated (Figure 4C). Tentative identification of the compounds can be found in Tables S2, Supporting Information. For example, VOC 48 (1‐tetradecanol) is elevated in PC mice model blood compared to naïve mice blood, while the same VOC is decreased in BC mice model blood compared to naïve mice blood. Some VOCs, significantly discriminate between all different cancer models, such as VOC 46 (Naphthalene derivative), while others discriminate between cancer models and naïve mice such as VOC 2 (2‐Butanone). Few VOCs also significantly altered between different stages of cancer, for example, VOC 15 (2‐Heptanone) that is significantly higher in BC mice model blood sample than in M‐BC mice model blood sample.

**Figure 5:**
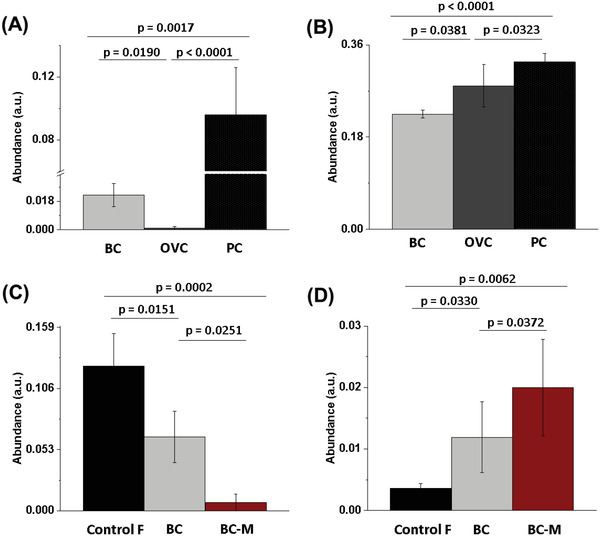
Statistically significant VOCs in the headspace of blood obtained from different cancer model mice as detected by the GC‐MS. A) VOC 14 (Unknown); B) VOC 46 (Naphthalene derivative); C) VOC 1 (2‐methyl‐2‐Propanol); D) VOC 39 (naphthalene derivative) abundance. BC‐breast cancer *n* = 36; M‐BC—metastatic breast cancer *n* = 10; OVC—ovarian cancer *n* = 21; PC—pancreatic cancer *n* = 39; F—female *n* = 22; M—male *n* = 36. NOTE: a.u. stands for arbitrary units of GC‐MS peaks AUC.

As seen in **Figure** **5**A, VOC 14 (unknown identification) is significantly lower in OVC mice blood than in BC and PC mice blood while showing higher abundance in PC mice blood with respect to BC mice blood (0.096 ± 0.03 a.u. in PC; 0.0011 ± 0.001 a.u. in OVC; 0.023 ± 0.007 a.u. in BC), while in Figure 5B we can see that VOC 46, a derivative of Naphthalene has the lower abundance in BC mice blood (0.224 ± 0.007 a.u.) and the higher in PC mice blood (0.32 ± 0.01 a.u.), and OVC in‐between (0.28 ± 0.04 a.u.). In addition, we can observe two potential metastatic biomarkers that showed significant differences between naïve, BC, and M‐BC mice models blood samples. VOC 1 (2‐methyl‐2‐propanol) abundance is decreasing as the disease stage advances (0.12 ± 0.02 a.u.; 0.06 ± 0.02 a.u.; 0.0072 ± 0.007 a.u., naïve, BC, and M‐BC respectively) while in contrast VOC 39 (1‐methyl Naphthalene) abundance is increasing as the disease progress (0.003 ± 0.0007 a.u.; 0.011 ± 0.005 a.u.; 0.02 ± 0.007 a.u., naïve, BC, and M‐BC respectively) (Figure 5C,D respectively).

## 3 Discussion

### 3.1. Analysis with the Artificially Intelligent Nanoarrays

Cancer biomarker field is rapidly advancing when more and more techniques are being developed to accurately identify and quantify them. For example, single‐molecule quantitative approaches, mainly optical or electrical technologies such as nanopore sequencing and optical mapping for epigenetic modifications;^[^ ^74^ ^]^ integrated microfluidics, quantitative phase imaging, and artificial intelligence (AI) on board “lab‐on‐chip” (LoC) for CTCs detection enumeration and characterization;^[^ ^75^ ^]^ Plasma metabolic fingerprint from uniform zeolitic imidazolate framework (ZIF‐8) crystals anchored onto FeOOH nanorods^[^ ^76^ ^]^ or, trimetallic alloys for fast, sensitive, and selective laser desorption/ionization mass spectrometry (LDI‐MS) analysis of small metabolites in serum with no pretreatment.^[^ ^77^ ^]^ In spite of these developments using highly selective sensing methods, only a limited number of indicating biomarkers for selected conditions, such as cancer VOCs, are available.^[^ ^78^, ^79^, ^80^, ^81^ ^]^ Though selective recognition in controlled setups and under confounding environmental factors is possible; the majority of diseases cannot be accurately diagnosed by VOC(s) alone.^[^ ^57^, ^58^, ^60^ ^]^ An additional challenge is the ability to synthesize highly selective probes or nanomaterials for each VOC, notably when they are nonpolar.^[^ ^82^ ^]^ Our results pave the way to overcome these challenges by using cross‐reactive (i.e., semi‐selective) nanotechnology‐based sensor arrays, using pattern recognition,^[^ ^83^, ^84^, ^85^ ^]^ viz. the adaptable approach of nanoarray.^[^ ^62^, ^63^, ^64^, ^65^, ^69^, ^87^, ^88^, ^89^, ^90^ ^]^ Arguably, the reported artificially intelligent nanoarray is better fit for fast diagnostic approaches in which assessment of a VOC collection is qualitative and semi‐quantitative. Selectivity is attained through pattern recognition of the collection.^[^ ^91^, ^92^ ^]^ Due to its cross‐reactivity nature, each sensor responds to a wide variety of VOCs, thus allowing detection of individual components from multi‐component samples.^[^ ^63^, ^70^, ^87^, ^88^, ^93^, ^94^ ^]^ Indeed, the basic principle of the artificially intelligent nanoarray is built on the capability of each sensor to detect all or some of the sample VOCs. While compared to selective sensors, the cross‐reactive sensors individually might suffer from an inferior sensitivity towards a definite VOC, however, they are much more flexible in sensing multi‐factor VOC mixtures in different environments.^[^ ^66^, ^86^, ^87^, ^88^, ^93^, ^94^, ^95^, ^96^, ^97^ ^]^

The binary classifiers based on sensing signals achieved very high accuracy in most cases, supported by the leave‐one‐out cross‐validation method for each classifier. To reduce the chance of overfitting the results a minimal number of sensors (2–3) was selected for each comparison. Moreover, applying the same classifiers to the corresponding control groups could easily reveal cases in which discrimination was based on artifacts/bias/confounding factors. While accuracy levels attained amongst diseases were high (86%), in one case the discrimination among the control samples gave an accuracy of 58% (i.e., random classification), while in another case classification reached 70% accuracy, this implies a possible bias related to mice model sex that should be ruled out in future experiments. Importantly, the sensors results back‐up the hypothesis that similar VOCs patterns are likely the result of resemblances in pathophysiological processes. The results also showed that the tuning for possible confounding factors interference was effective and probably less influenced by such factors.

These observations could be explained by the fact that the detection of specific VOCs biomarkers by the nanoarray does not depend on a lock‐and‐key approach. Indeed, the nanoarray depends on sensors that have diverse chemistries. In this situation, the VOCs emitted from blood are adsorbed onto the sensors’ surface. Specific VOCs combination could have a better affinity to the sensor's surface than others, due to the different chemistry of each sensor. In this way, the different combinations of VOCs could be detected by different sensors. By applying AI methods, the combination of these differences can be expressed and attributed to specific diseases.^[^ ^67^, ^98^, ^99^ ^]^

Recently we presented a new concept for liquid biopsy, based on alterations of VOCs profile in blood. We demonstrated in vitro that VOCs profile is significantly altered depending on the incidence and amount of cancer cells in the blood and can be easily detected by our sensor array.^[^ ^62^ ^]^ Here we presented an exploration study as a proof of concept that this can be achieved in a whole‐animal model and that blood VOCs pattern can discriminate effectively between healthy, cancer, and metastatic cancer mice models. Pushing toward a vision of early cancer diagnosis in the clinic in a minimally invasive, non‐expensive, or time‐consuming manner.

### 3.2. Analysis with the GC‐MS

In order to get insights into the chemical composition of the samples and possible unique biomarkers, samples were analyzed using GC‐MS as a gold standard system for volatile analysis. This step was used as an independent analytical tool to support the sensors’ results and to provide data that could serve in the next step to advance the sensing platform development. Indeed GC‐MS results showed that few VOCs could serve as potential biomarkers for cancer classification and staging. Over 200 VOCs were identified in each group, out of which 69 VOCs could discriminate between different cancers. These VOCs were tentatively identified and some were previously documented in the literature. For example, nonanal was linked to OVC^[^ ^100^ ^]^ and BC.^[^ ^101^ ^]^ As another example, 2‐Pentanone could be linked to pancreatic cancer (PC).^[^ ^102^ ^]^ Such data support our claim that no single VOC can classify amongst diverse cancers. This is in agreement with the fact that the majority of reported cancer biomarkers are not sensitive enough to serve as a single diagnostic test for current healthcare needs.

The clustering analysis that was done (Figure 4C) supports this claim and shows that considering a “pattern” based on different biomarkers rather than using just one, actually provides logical meaning for the data and provides information otherwise overseen. The BC clusters together with the M‐BC, which are indeed the most similar, as well as all three control subgroups cluster together. Therefore, the use of VOCs patterns in blood samples becomes a more realistic option for discriminating between different disease states. As seen in Figure 5C 2‐methyl‐2‐propanol may be considered a potential biomarker for BC diagnosis and staging. It was found that as the disease progresses in mice models, its levels are elevating. Previously naphthalene was also found as a potential biomarker for BC in humans,^[^ ^103^ ^]^ although with the opposite trend arises the limitation of the search for a specific biomarker that may change according to study settings or methods. The VOC, 2‐butanone was shown as a potential biomarker (sampled from urine) for hepatocellular carcinoma and is related to a number of processes in the body that are relevant to the malignant process, including inhibition of the membrane‐bound monoamine oxidase activity that is related to metastasis suppress by inhibiting the adrenergic system and its transactivation of epidermal growth factor receptor signaling.^[^ ^104^ ^]^ In addition, although the p‐value showed significant changes, the values discriminating between healthy and primary tumor stages are not satisfying. On the contrary, we were able to perceive a distinct pattern based on 69 VOCs for each cancer type, including a metastatic stage for one of the models. This implies that the use of a pattern rather than a single biomarker is more reliable. Single target‐specific sensing approaches are useful when there are one or two known biomarkers. However, diagnosis of such complicated and systemic disease as cancer requires observing hundreds of biomarkers, as seen in our GC‐MS results. Identified patterns may be further tested for possible concentrations and/ or combinations by means of sensors response and groups discrimination. This data also supports the use of “the pattern” concept for the liquid biopsy approach, examining the whole profile eliminates the need to develop specific methods or selective sensors for each biomarker and reduces the number of tests needed on each sample. This is true for other omics methods where the use of ML models for pattern recognition improved technique performance. For example, the use of sparse regression ML of patterns on direct serum metabolic patterns to diagnose early‐stage lung adenocarcinoma;^[^ ^105^ ^]^ or deep learning of Serum metabolic fingerprints (SMFs) and clinical indexes to provide enhanced diagnostic outcomes for stroke.^[^ ^106^ ^]^

Regression models applied to the raw GC‐MS data showed that the abundance of blood VOCs was affected by some common confounding factors. A number of VOCs were affected by gender (e.g., 1,3‐dimethyl Benzene, 2‐Heptanone, Heptacosane). This effect might be attributed to hormonal or structural gender‐related differences.^[^ ^107^ ^]^

## 4 Conclusions 

We have shown that cancer‐specific VOCs in mice models' blood samples can be accurately detected by our artificially intelligent array of cross‐reactive nanomaterial‐based chemical sensors. We were able to discriminate blood samples from different cancer models from naïve blood samples with >84% accuracy, >86% sensitivity, and >80% specificity with three features based DFA model. Discrimination accuracy reached over 93% when comparing different cancer models, indicating a potential detection and classification tool for cancer, emphasizing that each cancer has its unique pattern and does not affect the other. GC‐MS showed a distinguishing pattern of VOCs, depending on the cancer type and stage (primary tumor or metastatic), composed of 69 different compounds that were significantly different between the groups. The ability to detect cancer‐related VOCs patterns using our artificially intelligent nanoarray in small concentrations in a complex medium as blood by changing only mathematical models is promising and promotes developing of highly accurate early detection and monitoring tool for cancer. This non‐invasive monitoring method will grant physicians the ability to determine the exact stage of tumor development, allowing them to “tailor” the specific treatment that will lead to the optimal therapeutic results at that particular time.

## 5 Experimental Section

### Animal Models

All animal studies were approved by and complied with the institutional ethical committee at the Technion – Israel Institute of Technology (Breast and Ovarian approval #IL073517 and Pancreatic #IL1050815). Animal well‐being was monitored daily by the research team and the Technion Pre‐Clinical Research Authority veterinary staff.

### BC Tumor Model

As previously described by Krinsky et al.^[^ ^108^ ^]^ 50 µL of 6 × 10^6^ cells mL^−1^ of 4T1 overexpressing mCherry cell line were injected into 10‐week‐old BALB/c female mice (Harlan Laboratories Inc., Jerusalem, Israel); A BD Micro‐Fine plus 29G insulin syringe (BD, New Jersey, USA) was used to inject the cells into each mouse's fifth fat pad.

### PC (i.e., Pancreatic Ductal Adenocarcinoma ) Tumor Model

As previously described by Zinger et al.^[^ ^109^ ^]^ (10‐week‐old C57BL/6 mice (Harlan Laboratories, Jerusalem, Israel) were anesthetized using Ketamine/Xylazine (100 mg kg^−1^ and 10 mg kg^−1^ body‐weight, respectively) injected intraperitoneally. A subcutaneous injection of 0.05 mg kg^−1^ buprenorphine was performed before the surgery. The mice were placed on 37 °C warmed pads. The mice's lateral, abdominal left side was shaved, and a 1‐cm longitudinal cut was performed above the pancreas in the skin. While observing the spleen and the pancreas 1‐cm cut was done in the peritoneum. The pancreas was then secured using forceps. 250 000 KPC‐mCherry cells were suspended in 0.01 mL at 4 °C phosphate buffered saline (PBS) and injected using 5 µl Hamilton syringe with 30G at 30 degrees using a point style 4 needle (Hamilton, NV, USA) pancreas. Cuts were sutured using 5‐0 degradable sutures (Vicryl, AR, USA).

### OVC Tumor Model

As described by Poley et al.^[^ ^110^ ^]^ mouse ovarian surface epithelial (MOSE) cells. Ten‐week‐old C57BL/6 mice (Harlan Laboratories, Jerusalem, Israel) were anesthetized using Ketamine/Xylazine (100 mg kg^−1^ and 10 mg kg^−1^ body‐weight, respectively) injected intraperitoneally. A subcutaneous injection of 0.05 mg kg^−1^ buprenorphine was performed before the surgery. The mice were placed on 37 °C warmed pads. A 10 mm skin incision was made in the approximated location of the ovary. Additional 10 mm incision was made in the peritoneum. The fat pad surrounding the ovary was located and 10 µL of cell‐matrigel suspension containing 100 000 MOSE cells was injected directly into the ovarian bursa.

### In Vivo Imaging—Micro‐CT Scan

All mentioned tumors were imaged using an IVIS Spectrum CT Pre‐clinical in‐vivo imaging system (PerkinElmer, MA, USA) after placing the mice under isoflurane anesthesia. The following parameters were used to assess each type of tumor: BC‐ex. 570 nm em. 620 nm, binning 4, f‐stop 2, and exposure time 3 s. OVC‐ open emission filter, binning 16, f‐stop 383 1, and exposure time of 180 s, 12 min after IP injection of 150 mg kg^−1^ of D‐luciferin. PC: excitation 570 nm, emission 620 nm, binning‐ 4, f‐stop 2 at 3 s exposure.

### Blood Collection

Mice were euthanized using CO_2_. Approximately 1.2 mL of blood was drawn from the portal vein using a 1 cc syringe with 0.2 µL of 20 mM Tri‐sodium citrate used as an anticoagulant. Blood tubes were kept on ice at 4 °C until processed.

### Collection of Blood Headspace

Blood samples were transferred into headspace glass vials. The vials were closed for 1 h followed by heating the vial on a hot plate to 70 °C for 15 min prior to sampling. Then after, samples were pre‐concentration on Tenax TA tube (Sigma‐Aldrich) using clean N2 as a carrier gas together with hydrocarbon and humidity filter (super clean gas filters (SGT)) through stainless steel needles (5 cm, 14 g) at 100 mL min^−1^ for 15 min. The collected tubes were transferred to the thermal desorption (TD) system connected to the GC‐MS and the TD‐GC‐E‐Nose system.

Thermal desorber–gas chromatography–mass spectrometry (TD–GC–MS) A GC‐7890B/MS‐5977A instrument (Agilent) combined with a TD system (TD100; Markes International) was used. Pre‐desorption of the tubes lasted 2 min in a constant N_2_ flow of 20 mL min^−1^, followed by tube desorption under the following conditions: 8 min at 270 °C and 40 mL min^−1^ N_2_ flow. Cold‐trap desorption conditions were 3 min at 270 °C with N_2_ flow of 4 mL min^−1^. The samples were injected automatically from the TD into the GC‐system at constant pressure and under 2 mL min^−1^ column flow. The GC‐MS was equipped with an SLB‐5 ms capillary column (30 m length; 0.25 mm internal diameter; 1 µm thickness; Sigma‐Aldrich). The following temperature profile was applied: a) 2 min at 50 °C; b) 20 °C/min ramp until 300 °C; and c) 5 min at 300 °C. The MS mass range was set to 21–300 *m*/*z*. An internal standard mixture (EPA‐524) 1,4‐dichloro benzene‐D4 was added (20 ppb) along with all tested samples to ensure that the GC‐MS was functioning effectively.

### Intelligent Nanosensor Array

An array of 40 nanomaterial‐based sensors mounted on a customized polytetrafluoroethene circuit inside a stainless‐steel chamber was used (see Figure S3, Supporting Information). The sensors included gold‐nanoparticles (organically‐stabilized spherical Au nanoparticles, core diameter 3–4 nm) capped with different organic layers (tert‐dodecanethiol; butanethiol; 4‐chlorobenzenemethanethiol; 4‐*tert*‐butylbenzenethiol; dibutyl disulfide; 2‐naphthalenethiol; 2‐nitro‐4‐(trifluoromethyl)benzenethiol; dodecanethiol; octadecanethiol; decanethiol; 2‐ethylhexanethiol; 3‐ethoxythiophenol; benzylmercaptan; hexanethiol) as well as polymer‐coated 2D RN‐SWCNTs (diketopyrrolopyrole‐anthracene (FAF); diketopyrrolopyrole‐naphthalene (TNT); diketopyrrolopyrole‐benzothiadiazole (TBT); CB/poly(propylene‐urethaneureaphenyl‐disulfide) PPUU‐2S; CB/poly(propylene‐urethaneureaphenyl‐disulfide) PPUU‐2S mixed with poly(urethane‐carboxyphenyl‐disulfide) PUC‐2S (CB/(PUC‐2S/PPUU‐2S) composite); CB/poly(2‐hydroxypropyl methacrylate) mixed with polyethyleneimine (CB/(PPMA/PEI) Composite); crystal hexa‐perihexabenzocoronene (HBC) C12; hexyldecyl‐substituted poly(diketopyrrolopyrrole) (PDPPHD); polycyclic aromatic hydrocarbon 3 (PAH‐3)). Details regarding the fabrication and modification of the abovementioned sensors can be found in the literature.^[^ ^69^, ^70^, ^71^, ^111^ ^]^ The VOC samples were thermally desorbed at 270 °C in an auto‐sampler desorption system into a stainless‐steel sampling loop at 150 °C (TD20; Shimadzu Corporation, Japan). In parallel, the sensor chamber was kept under vacuum (≈30 mTor) until the introduction of the sample into the chamber. The remaining volume was filled with pure N_2_ to reach atmospheric pressure. resistance readings from the sensor array were measured using a Keithley data logger device (model 2701 DMM). The entire system was controlled by a custom‐made LabView program. The following sequence was used for each sample measurement: 5 min in vacuum, 5 min pure N_2_ gas, 5 min vacuum, 5 min sample or calibration exposure, followed by 5 min vacuum and 3 min pure N_2_ gas, and finally 3 min in vacuum. A fixed calibration gas mixture containing 11.5 ppm isopropyl alcohol, 2.8 ppm trimethylbenzene, and 0.6 ppm 2‐ethyl‐hexanol was exposed to the sensors daily to supervise their functionality during the experiment, and to overcome possible sensor response drift. Different features could be extracted from the sensor's signal, including Area under curve, delta R peak, delta R middle, and delta R end. The latter three features were based on the difference between the baseline resistance, during vacuum, and the resistance during the response to the sample exposure.

### Statistical Analysis—GC‐MS Data Processing

The GC‐MS chromatograms were analyzed using Mass Hunter qualitative analysis (version B.07.00; Agilent Technologies, USA). tentative identification of the VOCs was done using a spectral library match NISTL.14 (National Institute of Standards and Technology, USA), the Kruskal‐Wallis test, and an extension of the Non‐parametric Wilcoxon test, including Bonferroni alpha correction was used to identify significant differences in VOCs abundance between the groups. Hierarchical clustering using Ward's minimum variance method was applied. All data analyzed using SAS JMP, Verison.14.0 (SAS Institute, Cary, North Carolina, USA; 1989, 2005) was used for statistical analysis.

### Statistical Analysis—Discriminant Function Analysis (DFA)

Sensor's data were analyzed using DFA; it is a statistical method used when the groups to be discriminated are defined (labeled) before being analyzed, that is, predict a categorical outcome as a function of multiple continuous predictor variables.^[^ ^112^ ^]^ The input variables for DFA are the features extracted (as described above in nanoarray data) from sensors’ responses towards the headspace samples. The method applies dimension reduction of the data, it determines either a linear or quadratic combination of the input variables to receive minimum variance within each group and maximum variance between the groups to which new vectors, canonical variables (CV), are calculated. The decision on either linear or quadratic model was based on homogeneity of the variance‐covariance matrices of the tested groups according to statistical tests, for example, Bartlett's test. Leave‐one‐out cross‐validation was used to compute the success of the classification in terms of the numbers of true‐positive (TP), true‐negative (TN), false‐positive (FP), and false‐negative (FN) predictions. Given k measurements, the model was computed using k‐1 training vectors. All leave‐one‐sample‐out possibilities were considered, and the classification accuracy was assessed as the average performance over the k tests. Pattern recognition and data classification was performed using Python (Python Software Foundation). In addition, a ratio of 1:6 among samples and explanatory variables was maintained to reduce chance of overfitting.^[^ ^113^, ^114^ ^]^ Correct classification of the data points was counted and presented as sensitivity and specificity values according to Equations (1–5).

(1) $$\mathrm{Sensitivity}=\mathrm{TP}/\left(\mathrm{TP}+\mathrm{FN}\right)$$

(2) $$\mathrm{Specificity}=\mathrm{TN}/\left(\mathrm{FP}+\mathrm{TN}\right)$$

(3) $$\mathrm{Accuracy}=\left(\mathrm{TP}+\mathrm{TN}\right)/\left(\mathrm{TP}+\mathrm{TN}+\mathrm{FP}+\mathrm{FN}\right)$$

(4) $$\mathrm{Positive}\;\mathrm{predictive}\;\mathrm{value}\left(\mathrm{PPV}\right)=\mathrm{TP}/\left(\mathrm{TP}+\mathrm{FP}\right)$$

(5) $$\mathrm{Negative}\;\mathrm{predictive}\;\mathrm{value}\left(\mathrm{NPV}\right)=\mathrm{TN}/\left(\mathrm{TN}+\mathrm{FN}\right)$$

where TP = true‐positive, FN = false‐negative, TN = true‐negative, and FP = false‐positive.

## Conflict of Interest

The authors declare no conflicts of interest.

## Author Contributions

R.E.A. contributed to methodology, investigation provision of blood samples, data collection, formal analysis, and visualization. A.Z. contributed to tumor model formation and imaging, Y.Y.B. contributed to methodology, data curation, formal analysis, and manuscript revision. A.S. contributed to supervision of the project, and manuscript revision. H.H. was the coordinating principal investigator contributing to the conception, validation, supervision of the project, and manuscript revision. All authors read and gave their approval of this report.

## Supporting information

Supporting Information

## Data Availability

The data that support the findings of this study are available from the corresponding author upon reasonable request.
