# Supplementary material for: Artificially Intelligent Nanoarray Detects Various Cancers by Liquid Biopsy of Volatile Markers
Source: Adv Healthc Mater. 2022 Jul 14;11(17):2200356. doi: 10.1002/adhm.202200356 (PMC11468493; doi:10.1002/adhm.202200356)
Supplement: Supplementary file 1 — Supporting Information [file ADHM-11-2200356-s001.pdf]

ADVANCED HEALTHCARE MATERIALS

Supporting Information

for *Adv. Healthcare Mater.*, DOI 10.1002/adhm.202200356

Artificially Intelligent Nanoarray Detects Various Cancers by Liquid Biopsy of Volatile Markers

*Reef Einoch Amor, Assaf Zinger, Yoav Y. Broza, Avi Schroeder and Hossam Haick**

Supporting information

Artificially Intelligent Nanoarray Detects Various Cancers by Liquid Biopsy of Volatile Markers

*Reef Einoch Amor, Assaf Zinger, Yoav Y. Broza, Avi Schroeder, Hossam Haick**

R. Einoch Amor, Y.Y. Broza, H. Haick
Department of Chemical Engineering and Russell Berrie Nanotechnology Institute, Technion – Israel Institute of Technology, Haifa 3200003, Israel.
* Correspondence to: Hossam Haick (E-mail: hhossam@technion.ac.il)

A. Zinger, A. Schroeder
Laboratory for Targeted Drug Delivery and Personalized Medicine Technologies, Department of Chemical Engineering, Technion – Israel Institute of Technology, Haifa 3200003, Israel

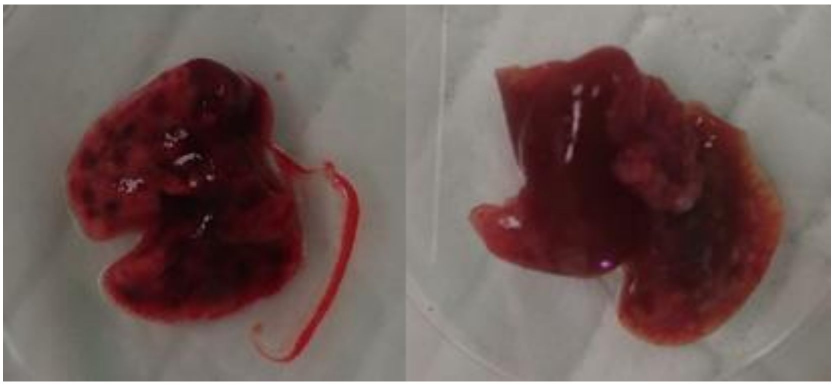

Figure S1. Representative image of metastatic lungs harvested from metastatic breast cancer mice model 14 weeks after primary tumor formation.

Table S1. List of sensors used for machine learning models, each model\comparison used two or three sensors

Comparison	1st Sensor	2nd Sensor	3rd Sensor
**BC vs. OVC**	CB/(PUC-2S/PPUU-2S) composite RN-SWCNTs	Butanethiol GNP's	PAH-3 RN-SWCNTs
**BC vs. PC**	Octadecanethiol GNP's	(HBC-C12) RN-SWCNTs	Hexanethiol GNP's
**OVC vs. PC**	Octadecanethiol GNP's	Benzylmercaptan GNP's	
**Control vs. OVC**	Decanethiol GNP's	2-Naphthalenethiol GNP's	
**Control vs. PC**	FAF RN-SWCNTs	3-Ethoxythiophenol GNP's	2-Nitro-4-(trifluoromethyl) benzenethiol GNP's
**Control vs. BC**	TNT RN-SWCNTs	4-Chlorobenzenemethanethiol GNP's	Hexanethiol GNP's
**BC vs. M-BC**	TNT RN-SWCNTs	2-Ethylhexanethiol GNP's	Octadecanethiol GNP's

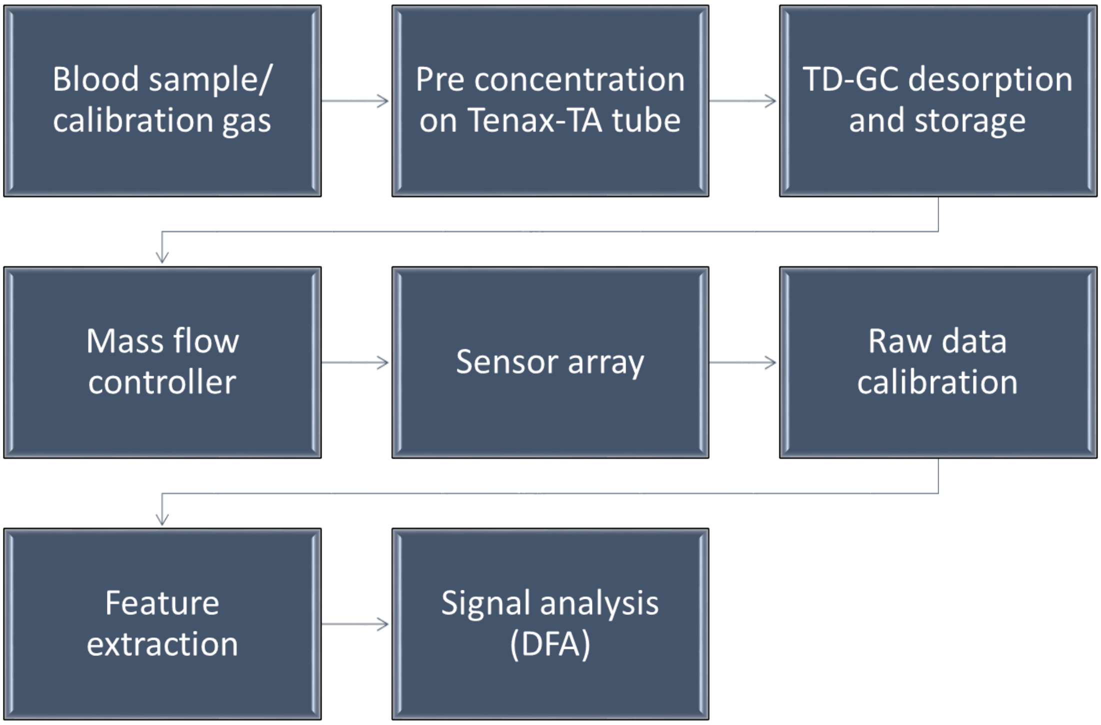

Figure S2: work flow chart for sensor array analysis

Sensor array fabrication, characterization and mechanism:

Blood headspace samples were analyzed using a sensor array containing 40 sensors based on two types of nanomaterial: organically-stabilized spherical gold nanoparticles (GNPs, core diameter: 3-4 nm), and Random Network of single-walled carbon nanotubes (RN-SWCNTs) see **Figure S3** for sensors characteristics. Different organic functionalities contributed to the chemical diversity of the sensors. The organic ligands provided broadly cross-reactive absorption sites for the blood VOCs.[1, 2]. Details regarding the fabrication and modification of the abovementioned sensors can be found in the literature.[3-7]

Each sensor in the array is not specific and designed to respond to several different chemicals [8-11]. A distinct pattern of the responses of a panel of arrays can provide a characteristic "fingerprint" of the vapor mixture, and allow classification and identification of the target mixture in the future. Mathematical algorithms for pattern recognition can be used for data analysis in these complex responses (e.g., Discriminant Factor Analysis (DFA)). These mathematical methods present multidimensional data in lower dimensions through emphasizing similarities and differences between the data [12-15]. The sensors combine a nonselective transducer with chemo-selective materials that serve as a vapor concentrator, resulting in a

highly sensitive detector that responds selectively to a particular class of chemical vapor. The electrical conductivity in chemiresistors based on monolayer-capped metallic nanoparticles (MCMNPs) occurs through the metallic cores, whereas the sorption of analyte molecules occurs through organic film coatings. The presence of well-defined organic spacers allows control over the inter-particle distance, thereby, obtaining a nearly uniform inter-particle distance in the composite films. This enables controlled signal and noise levels to be attained [16].

The sensing mechanism of the films can be affected by one or both of the following mechanisms: Swelling - This effect is based on the reversible swelling of the material upon gas absorption, which increases the spacing between the metal cores, thus to an increase in resistivity. Since the typical electron hopping conductivity in these materials is highly dependent on inter-particle distance, adsorption of organic vapors strongly decreases electrical conductivity. This is true for all thin layers where the swelling occurs in both horizontal and vertical directions.[17]; Permittivity- An increase in permittivity of the organic matrix surrounding the metal cores decreases resistance due to decreases in the activation energy and the tunneling constant. Hence, analytes with high dielectric constants (e.g., water, methanol) decrease resistance, whereas analytes with low dielectric constants (e.g., toluene, n-hexane) increase it [18].

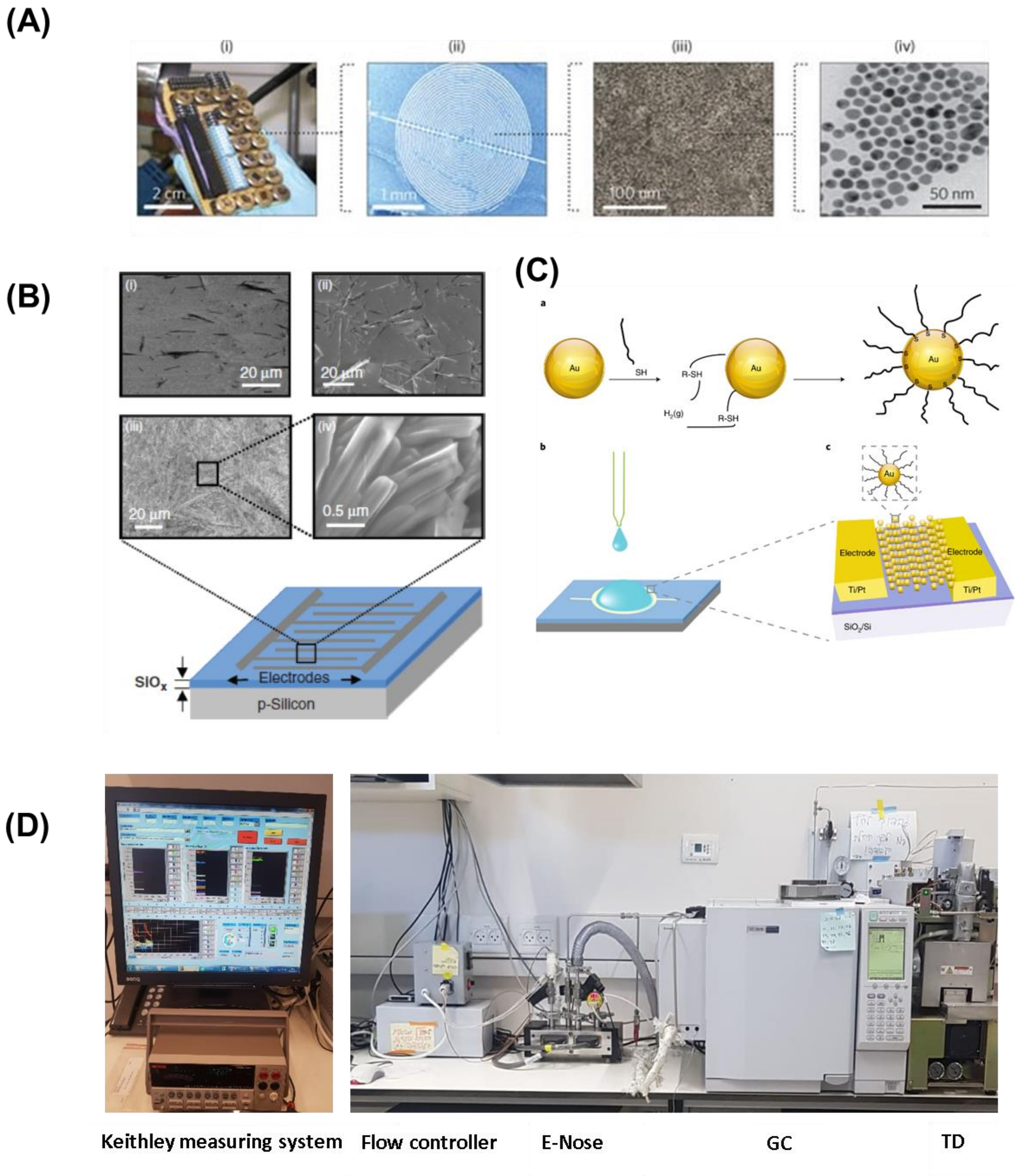

Figure S3. (A) Photograph, microscopy image, SEM, and TEM of the GNP-based sensors. Reproduced with permission from ref 4. Copyright 2009 Springer Nature Publishing.(B) (a) Schematic representation of a chemiresistor based on random networks of carbon nanotubes (RN-CNTs) that are partially covered by hexa-peri-hexabenzocorone-C12 layers (HBC-C12), as well as scanning electron micrographs of RN-CNTs covered by different portions with HBC-C12 layers. This was achieved by drop casting solutions with three different HBC-C12 concentrations, whereby a higher concentration in solution yielded a higher surface coverage: (1) 10−3 M HBC-C12 resulted in 10% surface coverage of HBC-C12; (2) 10−4 M in 30%; and (3) 10−5 M in 90%. The surface coverage was determined through digital image processing

from the different contrasts of covered and uncovered areas in the scanning electron micrographs; (4) shows a higher magnification of the 90% fi lm coverage. Note that the RN-CNT is not visible at the relatively low magnification used to show the HBC-C12 fi lms. Reproduced with permission from ref 1. Copyright 2010 MRS Bulletin (C) a, MCGNP synthesis by Brust method42. b, Inkjet printing above interdigital electrodes. c, MCGNP on the surface after drying. Reproduced with permission from ref 7. Copyright 2021 Nature Protocols. (D) TD-GC-E-Nose system.

VOCs in blood are at ultralow concentrations, such as ppm−ppt level, therefore sensors sensitivity should be high with low limit of detection (LOD) values. Analytical data with regards to the metal nanoparticles sensing sensitivity can be seen in different publications both from our group and some other studies. For example: In the study of VOCs from skin for TB detection sensors were evaluated and showed responses between 100 ppb and 140 ppm depending on the tested VOC[19], in another study[20] authors show sensitivity to low concentrations down to 50 ppb. Other studies reached also LOD of 8.8 ppt for toluene.[21] To reach these values of sensitivity the signal to noise ratio (SNR) should be high. We calculated the SNR by dividing the average resistance of the response curve by the standard deviation of the baseline resistance. A representative image of sensor response to headspace can be seen in **figure S4**. In **figure S4.A** we can see sensor response with high SNR value (125,000) while in **figure S4.B** we can see sensor response with low SNR value (350). Sensors with SNR value lower than 1000 where not included in the analysis.

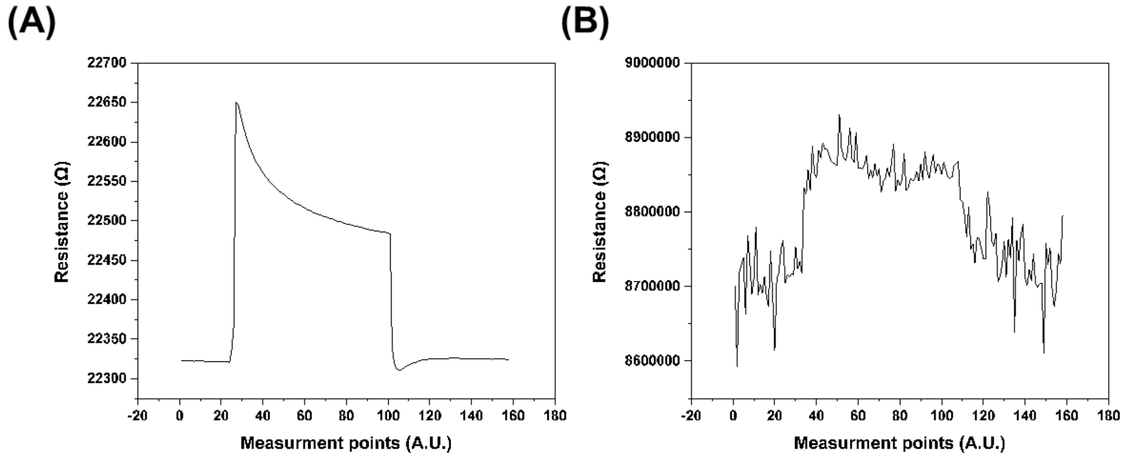

Figure S4: Representative response of (A) 3-Ethoxythiophenol GNP's sensor and (B) Tert-Dodecanethiol GNP's to blood headspace sample.

Table S2. List of significantly changed VOCs obtained from the headspace of blood from breast cancer, ovarian cancer, pancreatic cancer and control mice blood.

	RT	BC vs. M-BC	BC vs. PC	BC vs. OVC	PC vs. OVC	OVC vs. Control F	BC vs. Control F	M-BC vs. Control F	PC vs. Control M	Tentative Compound
1	3.02813	x					x	x		2-Propanol, 2-methyl
2	4.41932		x		x	x	x	x	x	2-Butanone or Acetic acid
3	5.32692								x	Chloroform
4	5.57897		x				x	x		Acetic acid
5	6.98432							x		Benzene
6	7.27123		x				x	x		1-Butanol
7	8.49178		x		x		x	x	x	2-Pentanone
8	11.74615			x			x	x		Isopentenyl alcohol (3-Buten-1-ol, 3-methyl-)
9	13.9628					x	x	x		1-Pentanol
10	15.36862		x						x	Hexanal
11	17.33993					x			x	Unknown
12	17.85812					x				ethyl- Benzene
13	18.3017					x			x	1,3- dimethyl Benzene
14	18.63568		x	x	x				x	Unknown
15	18.81352	x	x		x	x	x			2- Heptanone
16	19.00443								x	1,2- dimethyl Benzen/ p-Xylene/ Styrene
17	19.12032						x			2-methyl- cyclopentanone
18	21.2485							x		Benzaldehyde
19	21.55548		x		x				x	1-Octen, 3-ol/ Phenol/ Carbamic acid, methyl-, phenyl ester
20	21.98548			x	TBD	x			x	Heptane, 2,2,4,6,6-pentamethyl-
21	22.12162					x			x	1,2,4/ 1,3,5- trimethylbenzene
22	22.35353	x	x		x			x	x	1,3,5- trimethylbenzene
23	22.78372		x		x	x	x	x	x	4-cyanocyclohexene
24	22.91308			x	TBD	x		x		2-ethyl 1-hexanol
25	23.49292	x						x		Benzeneacetaldehyde
26	23.58157		TBD		TBD					Dodecane/ 2-butyl, 1-octanol
27	23.89563	x	TBD		TBD			x	x	2-octen, 1-ol (z)
28	24.07262			x		x				Acetophenone
29	24.68692					x				Dodecane/ Undecane
30	24.83027					x	x	x		Nonanal
31	26.94463		TBD						x	Dodecan/ Tridecane
32	27.16987					x	x			Decanal
33	28.11105		x	x		x		x	x	Benzothiazole
34	28.48633	x	x	x			x			Tetradecane, 1-chloro
35	28.51355		x							1-Decene/ Cyclododecane
36	28.77287	x				x		x	x	Unknoun
37	29.09347					x				Tridecane/ 1-Octadecanesulphonyl chloride/ 1-chloro Octadecane
38	29.51633		x							2-Ethylbutyric acid, eicosyl ester
39	29.59138	x					x	x		Naphtalene, 1-methyl
40	30.17853	x			TBD			x	x	2,3-dichloro- Benzenamine
41	30.33493		x		x		x			Tricycloundec-9-ene, 2,6,6,9-

										tetramethyl-/ (+)-Aromadendrene
42	30.45782		x		x	x	x	x		1H-3a,7-Methanoazulene, 2,3,6,7,8,8a-hexahydro-1,4,9,9-tetramethyl-, (1.alpha.,3a.alpha.,7.alpha.,8a.beta.)-
43	30.57367		x		x				x	1H-Cycloprop[e]azulene, 1a,2,3,4,4a,5,6,7b-octahydro-1,1,4,7-tetramethyl-, [1aR-(1a.alpha.,4.alpha.,4a.beta.,7b.alpha.)]-
44	30.75122	x	x	x			x	x		Unknown
45	31.09225					x				Tetradecane
46	31.80887	x	x	x	x			x	x	(8R,8aS)-2-Isopropylidene-8,8a-dimethyl-1,2,3,7,8,8a-hexahydronaphthalene
47	31.94497		TBD				x	x		4,4-Dimethyl-3-(3-methylbut-3-enylidene)-2-methylenebicyclo[4.1.0]heptane
48	32.1496	x	x	x			x			1-Tetradecanol
49	32.51787		x							5-Hydroxymethyl-1,1,4a-trimethyl-6-methylenedecahydronaphthalen-2-ol/ 1-chloro Hexadecane
50	32.89998			x		x				Pentadecane
51	33.1251					x	x	x		3,5,11-Eudesmatriene
52	33.20005						x			Phenol, 2,4-bis(1,1-dimethylethyl)-
53	33.39105	x								alpha-Patchoulene
54	33.51402					x	x			Tetradecane, 2,6,10-trimethyl-/ Docosane, 11-decyl-
55	33.73208		TBD	x		x				unknown
56	34.2985							x		1H-Cycloprop[e]azulene, 1a,2,3,5,6,7,7a,7b-octahydro-1,1,4,7-tetramethyl-, [1aR-(1a.alpha.,7.alpha.,7a.beta.,7b.alpha.)]-
57	34.38745	x	x		x			x		1,5,9 trimethyl cyclododecatriene
58	34.97378	x	x	x		x	x	x		Unknown
59	35.25347	x		x	TBD		x		x	secobarbital
60	36.23565						x			dotriconate
61	36.37225					x	x			[1,1'-Biphenyl]-2-ol, 5-(1,1-dimethylethyl)-
62	36.53597					x	x	x		Oleic acid
63	36.74745	x				x		x		Benzenesulfonamide, N-butyl-
64	36.76822				x	x	x	x		Unknown
65	37.76388	x		x	TBD	x		x		Unknown
66	38.21417			x	TBD	x	x	x		Heptacosane
67	38.5619						x			Unknown
68	40.33555	x				x	x	x		Heptacosane
69	41.05192					x	x	x		Heptacosane

[a)] BC-breast cancer; M-BC- metastatic breast cancer; OVC- ovarian cancer; PC-pancreatic cancer; F-female; M- male.

[b)] Significantly different VOCs ($p < 0.05$) based on averaged peak area (BC, n = 36; M-BC, n = 10; OVC, n = 17; PC, n = 38; Control F, n = 22; Control M, n = 34).

[c)] TBD- to be determined. May be an effect of male female differences

References

1. Tisch, U.; Haick, H. Nanomaterials for Cross-Reactive Sensor Arrays. *MRS bull.* **2010,** *35* (10), 797-803.
2. Tisch, U.; Haick, H. Sensors Based on Monolayer-Capped Metal Nanoparticles. *Chemical sensors* **2010,** *2*, 141-202.
3. Huynh, T.-P.; Khatib, M.; Srour, R.; Plotkin, M.; Wu, W.; Vishinkin, R.; Hayek, N.; Jin, H.; Gazit, O. M.; Haick, H. Composites of Polymer and Carbon Nanostructures for Self-Healing Chemical Sensors. *Adv. Mater. Technol.* **2016,** *1* (9), 1600187.
4. Peng, G.; Tisch, U.; Adams, O.; Hakim, M.; Shehada, N.; Broza, Y. Y.; Billan, S.; Abdah-Bortnyak, R.; Kuten, A.; Haick, H. Diagnosing Lung Cancer in Exhaled Breath Using Gold Nanoparticles. *Nat. nanotechnol.* **2009,** *4* (10), 669-673.
5. Dovgolevsky, E.; Tisch, U.; Haick, H. Chemically Sensitive Resistors Based on Monolayer-Capped Cubic Nanoparticles: Towards Configurable Nanoporous Sensors. *Small* **2009,** *5* (10), 1158-1161.
6. Zilberman, Y.; Ionescu, R.; Feng, X.; Müllen, K.; Haick, H. Nanoarray of Polycyclic Aromatic Hydrocarbons and Carbon Nanotubes for Accurate and Predictive Detection in Real-World Environmental Humidity. *ACS Nano* **2011,** *5* (8), 6743-6753.
7. Milyutin, Y.; Abud-Hawa, M.; Kloper-Weidenfeld, V.; Mansour, E.; Broza, Y. Y.; Shani, G.; Haick, H. Fabricating and Printing Chemiresistors Based on Monolayer-Capped Metal Nanoparticles. *Nat. Protoc.* **2021,** *16* (6), 2968-2990.
8. Elghanian, R.; Storhoff, J. J.; Mucic, R. C.; Letsinger, R. L.; Mirkin, C. A. Polynucleotides Based on the Distance-Dependent Optical Properties of Gold Nanoparticles. *Science* **1997,** *277*, 1078.
9. Gopel, W. Nanosensors and Molecular Recognition. *Microelectron. Eng.* **1996,** *32*, 75.
10. Gopel, W. Chemical Analysis and Sensorics with Microstructured Devices. *Mikrochim. Acta.* **1997,** *125*, 179.
11. Schierbaum, K. D. Application of Organic Supramolecular and Polymeric Compounds for Chemical Sensors. *Sens. Acta.B.* **1994,** *18*, 71.
12. Duda, R. O.; Hart, P. E. Pattern Classification and Scene Analysis. *John Wiley & Sons: New York* **1973**.

13. Geladi, P.; Kowalski, B. R. Partial Least-Squares Regression: A Tutorial. *Anal. Chim. Acta.* **1986,** *185*, 1-17.
14. Kowalski, B. R.; Bender, C. F. K-Nearest Neighbor Classification Rule (Pattern-Recognition) Applied to Nuclear Magnetic-Resonance Spectral Interpretation. *Anal. Chem.* **1972,** *44* (8), 1405.
15. Martin, M.; Santos, J.; Agapito, J. Application of Artificial Neural Networks to Calculate the Partial Gas Concentration in a Mixture. *Sens. Acutat. B.* **2001,** *77*, 468.
16. Zhang, J. Z. Ultrafast Studies of Electron Dynamics in Semiconductor and Metal Colloidal Nanoparticles: Effects of Size and Surface. *Acc. Chem. Res.* **1997,** *30*, 423-429.
17. Steinecker, W. H.; Rowe, P. M.; Zellers, T. E. Model of Vapor-Induced Resistivity Changes in Gold-Thiolate Monolayer-Protected Nanoparticle Sensor Films. *Anal. Chem.* **2007,** *79*, 4977-4986.
18. Joseph, Y.; Guse, B.; Vossmeyer, T.; Ysuda, A. Gold Nanoparticle/Organic Network as Chemiresistor Coatings: The Effect of Film Morphology on Vapor Sensitivity. *J. Phys. Chem. C* **2008**.
19. Vishinkin, R.; Busool, R.; Mansour, E.; Fish, F.; Esmail, A.; Kumar, P.; Gharaa, A.; Cancilla, J. C.; Torrecilla, J. S.; Skenders, G. Profiles of Volatile Biomarkers Detect Tuberculosis from Skin. *Adv. Sci.* **2021,** *8* (15), 2100235.
20. Kahn, N.; Lavie, O.; Paz, M.; Segev, Y.; Haick, H. Dynamic Nanoparticle-Based Flexible Sensors: Diagnosis of Ovarian Carcinoma from Exhaled Breath. *Nano lett.* **2015,** *15* (10), 7023-7028.
21. Hanwell, M.; Heriot, S.; Richardson, T.; Cowlam, N.; Ross, I. Gas and Vapour Sensing Characteristics of Langmuir-Schaeffer Thiol Encapsulated Gold Nanoparticle Thin Films. *Colloids Surf. A* **2006,** *284*, 379-383.
